# Supplementary material for: A reinforcement learning approach to improve the performance of the Avellaneda-Stoikov market-making algorithm
Source: PLoS One. 2022 Dec 20;17(12):e0277042. doi: 10.1371/journal.pone.0277042 (PMC9767337; doi:10.1371/journal.pone.0277042)
Supplement: Attachment — Submitted filename: rebutal_letter - Alpha-AS - PLOS ONE - 2022.docx [file pone.0277042.s001.docx]

10^th^ April 2022

Dear reviewers,

We would like to submit a completely new version based on your concerns of the previous one.

We have taken the same idea, but we have rewritten completely the document changing the title, structure, experiments and how we present the results.

We appreciated so much your comments and take the main ideas to improve it.

1. *Is the manuscript technically sound, and do the data support the conclusions?*
   We have completely rewritten the document
2. *Has the statistical analysis been performed appropriately and rigorously?*
   We have included more tests (30 days) and a statistical analysis have been performed on this results
3. *Is the manuscript presented in an intelligible fashion and written in standard English?*

We have completely rewritten the document

There most important point for us were the comments for the author section, that we can conclude the same ideas as before. The previous version of the document was a good idea to research with interesting results, but the way we exposed these ideas and expressions were poor.

We have written a new version with the same ideas but the way we have exposed them in better, technical way.

We hope you like this new version!

Yours faithfully,

Javier Falces Marin

Universidad Politécnica de Madrid (UPM)

Reviewers' comments:

Reviewer's Responses to Questions

**Comments to the Author**

1. Is the manuscript technically sound, and do the data support the conclusions?

The manuscript must describe a technically sound piece of scientific research with data that supports the conclusions. Experiments must have been conducted rigorously, with appropriate controls, replication, and sample sizes. The conclusions must be drawn appropriately based on the data presented.

Reviewer #1: Yes

Reviewer #2: Partly

Reviewer #3: No

2. Has the statistical analysis been performed appropriately and rigorously?

Reviewer #1: Yes

Reviewer #2: I Don't Know

Reviewer #3: No

3. Have the authors made all data underlying the findings in their manuscript fully available?

The [PLOS Data policy](http://www.plosone.org/static/policies.action#sharing) requires authors to make all data underlying the findings described in their manuscript fully available without restriction, with rare exception (please refer to the Data Availability Statement in the manuscript PDF file). The data should be provided as part of the manuscript or its supporting information, or deposited to a public repository. For example, in addition to summary statistics, the data points behind means, medians and variance measures should be available. If there are restrictions on publicly sharing data—e.g. participant privacy or use of data from a third party—those must be specified.

Reviewer #1: Yes

Reviewer #2: Yes

Reviewer #3: No

4. Is the manuscript presented in an intelligible fashion and written in standard English?

PLOS ONE does not copyedit accepted manuscripts, so the language in submitted articles must be clear, correct, and unambiguous. Any typographical or grammatical errors should be corrected at revision, so please note any specific errors here.

Reviewer #1: No

Reviewer #2: Yes

Reviewer #3: No

5. Review Comments to the Author

Please use the space provided to explain your answers to the questions above. You may also include additional comments for the author, including concerns about dual publication, research ethics, or publication ethics. (Please upload your review as an attachment if it exceeds 20,000 characters)

Reviewer #1: This paper presents a new technique for market making based on deep reinforcement learning. Then, this approach was applied to the real market dataset. In this reviewer’s opinion, the idea of using deep reinforcement learning for market making is with limited innovation. Some comments are listed below.
1. It's strange that there are too many paragraphs in this paper.
2. The introduction of recent research is not enough. In particular, the progress about reinforcement learning based method needs further introduction.
3.The proposed method consists of several parts. It is suggested to analyze the effect of each part on improving the accuracy.
4. The experimental results are not convincing. The author should explain how to choose algorithms to evaluate the proposed method.

Reviewer #2: The study seems interesting and sound. Undoubtedly, reinforcement learning and cryptocurrencies are both hot topics in their own ways and mertits. Nevertheless, I consider the manuscript is not writing at the research/academic level and consequently most of the results go unnoticed and do not attract the attention of the reader. Please rewrite the manuscript more formally with an appropriate structure of ideas, paragraphs, equations, and citations. Particularly, improve the review of literature section, description of the results and their implications. The methodology must be described mathematically. Please consider resubmit.

Reviewer #3: The introduction contains several basic material about market microstructure that could be omitted or better presented (I suggest to read Foucault, T., Pagano, M., Roell, A., & Röell, A. (2013). Market liquidity: theory, evidence, and policy. Oxford University Press.). Nevertheless, in general the goal of the paper is not clearly stated. For example, only at the end the authors state "In this research we are going deep into machine learning techniques to optimize, train and create algorithms that are able to perform better than classical mathematical models.", that is too general and not enough clear.

In the section 2 the algorithm is presented. Despite sometimes a not-academic jargon is used (e.g "it is something in between"), in general all the material (included formulas) is poorly presented and difficult to follow. The material of this section must be better organized following similar studies (some have been also included in the reference section of the paper).

The section 3 contains the data used for the application. What is the "btc-usdt of December 2020" dataset? The data used must be better presented, with some descriptive statistics and much more details. The classification steps based on Hurst exponent are unclear too. What does it mean "It’s a random walk that can be classified using Hurst exponent where..." what is a random walk? The considered stock? It is unclear.

Even if the underlying idea of the paper could be in principle interesting, the work is presented in an inappropriate way. The English is too poor, the paper structure is weak and the goal unclear.

6. PLOS authors have the option to publish the peer review history of their article ([what does this mean?](https://journals.plos.org/plosone/s/editorial-and-peer-review-process#loc-peer-review-history)). If published, this will include your full peer review and any attached files.


If you choose “no”, your identity will remain anonymous but your review may still be made public.


**Do you want your identity to be public for this peer review?** For information about this choice, including consent withdrawal, please see our [Privacy Policy](https://www.plos.org/privacy-policy).

Reviewer #1: No

Reviewer #2: No

Reviewer #3: No
