# Supplementary material for: A reinforcement learning approach to improve the performance of the Avellaneda-Stoikov market-making algorithm
Source: PLoS One. 2022 Dec 20;17(12):e0277042. doi: 10.1371/journal.pone.0277042 (PMC9767337; doi:10.1371/journal.pone.0277042)
Supplement: Attachment — Submitted filename: Alpha-AS - PLOS ONE submission - Responses to reviewers.docx [file pone.0277042.s002.docx]

Javier Falces Marín

Universidad Politécnica de Madrid

Avenida Complutense, 30 – Dcho C-304.1

28040 Madrid

Spain

J. E. Trinidad Segovia

Section Editor

PLOS ONE

9^th^ July 2022

PLOS ONE *paper Ref.:* **PONE-D-22-10287**

*Title:*

“A reinforcement learning approach to improve the performance of the Avellaneda-Stoikov market-making algorithm”

*Dear Ms. Trinidad Segovia and reviewers,*

*I would like to thank you and the reviewers for your help in preparing this paper. We have followed the advice and suggestions given by the reviewers, and feel the article has benefited substantially from them. We hope we have succeeded in improving the paper to the reviewers’ and the editors’ satisfaction.*

*The following pages of this document contain our replies to the reviewers’ comments, explaining how we have addressed the corresponding issues in our revision of the paper.*

*Thank you for your interest in our research and for considering the possibility of publishing our work.*

*Kind regards,*

*Javier Falces Marín*

**Responses to the reviewers’ comments**

Comments from the reviewers are on grey background.

Our responses are on white background.

**Additional Editor Comments:**

In view of the referees’ feedback and my own reading of your paper, I invite you to address all issues noted below. I consider these issues to be major in nature, requiring more than a superficial or minor revision. In particular, there are important deficiencies in the methodological section that seriously hinder the understanding of the work as well as the results obtained. Their robustness is also unclear, so I have doubts as to whether the conclusions are supported by the results presented.

We hope the amendments to the paper and our responses to the reviewers answer your concerns. We certainly feel the reviewers’ comments and suggestions have helped us improve the paper significantly. For this we are grateful.

**Comments from REVIEWER #1**

1- What about running time (execution time) of the method?

We have added the following line to section 5.2 (“Training”):

“The training time for each Alpha-AS model was approximately 7 hours.”

2- The "Related Work" section is missing. A new section should be added between "Introduction" and "Section 2". Some parts of the "Introduction" section can be moved to the new section.

We have reorganised the contents of sections 1, 2 and 3. Section 2 has been renamed to “Background: The Avellaneda-Stoikov procedure”. Section 3 has been renamed to “Related work on machine learning in trading”.

We consider that this structure suits our paper best, since we provide background and related work for both AS and machine learning as they relate to trading. We also note that PLOS ONE’s submission guidelines do not require a specific “Related Work” section (https://journals.plos.org/plosone/s/submission-guidelines).

In addition, providing a table that summarizes the related work would increase the understandability of the difference from the previous studies in the related works section.

This is a good suggestion. We do not attempt to provide a comprehensive overview of related studies, however, as there are many, with various approaches, complementary or overlapping. Instead of a table, we have added references to works that do provide very good overviews of the work in the field, particularly:

Gašperov, Bruno, and Zvonko Kostanjčar. "Deep Reinforcement Learning for Market Making Under a Hawkes Process-Based Limit Order Book Model." IEEE Control Systems Letters (2022).

Patel, Yagna. "Optimizing market making using multi-agent reinforcement learning." arXiv preprint arXiv:1812.10252 (2018).

3- There are many symbols. A notation table can be added to give all symbols and their meanings.

This is a useful idea. Thank you. We have added a table of symbols.

4- The symbols in the text should be italic.

For example:

- "The features are as follows (with 0 ≤ X ≤ 4)"

- "the agent defines 5 features (N = 5)"

- ... etc.

We have changed the formatting of these symbols to italic.

**Comments from REVIEWER #2**

The paper is generally well written and in my opinion above the threshold for publication, however, there are certain issues that should be addressed prior to it. Therefore I recommend the manuscript to be accepted pending minor revision.

We thank the reviewer for their positive assessment.

Grammar/spelling/other:

1) Page 4 => "Section2" should be replaced by "Section 2", "making trading (3, 8) ." should be replaced by "making trading (3, 8)."

2) Please proofread the whole manuscript once again

3) Page 7=> "o" should be changed to "of"

4) Page 8 => "The [(, ) + max ′ (′, ′)] is referred to as the target Q-Value" => add the word "TERM" before "is referred"

5) Page 19 => "Error! Reference source not found.Table" => please amend this

Thank you for pointing out these issues. We have fixed them and we have proofread the manuscript. Nevertheless, further typos, formatting and other problems are sure to remain. Should the reviewers find more in the new version of the manuscript, we hope they are kind enough to point them out to us!

6) "Linear in Inventory with Inventory Constraints (Linearly constant spread) (10): LIIC is also a constant linear spread algorithm that places first-level quotes on both sides of the market. It differs from FOIC in its inventory offset strategy to reduce risk: in LIIC, when an positive (negative) inventory threshold is reached, the quantity of the buy (sell) orders is decreased linearly." => this is false, that is not how the LIIC works. LIIC does not place first-level quotes on both sides of the market but rather determines the offsets as a linear function of the current inventory. Please change this.

The reviewer is right to point out that our original working was deficient. The paragraph now reads:

“LIIC is also a constant linear spread algorithm that can place first-level quotes on both sides of the market. It differs from FOIC in its inventory offset strategy to reduce risk: in LIIC the quantity of the buy (sell) orders is decreased linearly as the positive (negative) inventory increases. When a positive (negative) inventory threshold is reached, buy (sell) orders are interrupted.”

Literature review:

1) I recommend also referencing the following papers:

Guéant, O. (2017). Optimal market making. Applied Mathematical Finance, 24(2), 112-154.

- provides a thorough overview of market making

Gašperov, Bruno, et al. "Reinforcement Learning Approaches to Optimal Market Making." Mathematics 9.21 (2021): 2689.

- provides a survey of RL approaches to market making

Patel, Yagna. "Optimizing market making using multi-agent reinforcement learning." arXiv preprint arXiv:1812.10252 (2018).

- application of multi-agent RL to market making

Gašperov, Bruno, and Zvonko Kostanjčar. "Deep Reinforcement Learning for Market Making Under a Hawkes Process-Based Limit Order Book Model." IEEE Control Systems Letters (2022).

These are very pertinent references. We have added them to the paper. We thank the reviewer for these contributions.

- consideration of more sophisticated limit order book and market making models

Comments:

1) Page 5 => explain that the Poisson rate at which the orders are executed at a Poisson rate decreases linearly with the spread

This is an assumption present in the derivation of the AS method, in Avellaneda and Stoikov’s original paper. We simply report it as is, along with the other main assumptions of the approach. To clarify a little more, we have added the following rationale behind the assumption (here in italics):

“The models underlying the AS procedure, as well as its implementations in practice, rely on certain assumptions. Statistical assumptions are made in deriving the formulas that solve the P&L maximization problem. First, it is assumed that the agent’s orders are executed at a Poisson rate which decreases as the spread increases *(i.e., the farther away from the market mid-price an order is placed at, the more time should tend to elapse before it is executed)*; second […]”

2) Page 6 => "The AS model generates bid and ask quotes that are optimal only if all of the assumptions it relies on are accurate." => not really, this statement is too strong - the AS model ignores the existence of the bid-ask spread and is ill-suited for market making (MM) on stocks and better suited for MM on quote-driven markets (bonds etc.) Elaborate on this a bit more.

We agree with the reviewer and we have rephrased accordingly. We have added text to clarify (hopefully) that our point is simply to remark on the deviations of from optimality of the AS models when working in real trading environments:

“The AS model generates bid and ask quotes that aim to maximize the market maker’s P&L profile for a given level of inventory risk the agent is willing to take, relying on certain assumptions regarding the microstructure and stochastic dynamics of the market. Extensions to the AS model have been proposed, most notably the Guéant-Lehalle-Fernandez-Tapia approximation (11), and in a recent variation of it by Bergault et al. (49), which are currently used by major market making agents. Nevertheless, in practice, deviations from the model scenarios are to be expected. Under real trading conditions, therefore, there is room for improvement upon the orders generated by the closed-form AS model and its variants.”

(We do not discuss what markets are best suited for market making, as we consider this falls beyond the scope of the paper. Nevertheless, we are willing to reconsider if the reviewer argues for the need to do so.)

3) Page 10 => "last N ticks (orders entered in the order book)." Why do you only mention order arrivals here, and not cancelations as well, since they also change the state of the limit order book? Add cancelations as well.

The reviewer is right to mention cancellations. We have rephrased the text to reflect them:

“the last *N* ticks (orders entered in the order book, as well as cancellations)”

We have also added a brief clarification of our considerations behind our choice of values for N:

“In each case, a value of *N* was chosen large enough to provide the agent with a sufficiently rich state space from which to learn, while also small enough that training demands a manageable amount of time and resources.”

Questions:

1) The authors rely on the use of a genetic algorithm to determine the parameters of the AS formulas. Why are the advantages of using this as compared to simply calibrating the parameters, for example in accordance with this procedure laid out here: https://www.theses.fr/2015PA066354.pdf (PhD thesis by Joaquin FERNANDEZ TAPIA). Please elaborate on it in the body of the manuscript.

Fernández-Tapia’s closed-form solution to parameter calibration is a very worthy theoretical approach, which we will certainly consider in future work, which may include comparative tests (as is now reflected in the Conclusions). In our present work we have simply chosen to follow the more practically oriented genetic algorithm route, which is grounded on data. We have added the following paragraph (Section 4.2):

“The size of this parameter space is large, and we need to find the values that make the AS model perform as close to optimally as possible. One way to achieve this would be to calibrate the parameters using closed formulas derived from reasonable statistical models, in the line explored by Fernández-Tapia (REF). Another option is to rely on genetic algorithms, which have been applied widely to calibrate machine learning models (36–38). In algorithmic trading they are commonly used to find the parameter values of a trading model that produce the highest profit (39). This motivated us to lean on a genetic algorithm to find the best-performing values for our parameters (35).”

2) Page 8 => "actions performed by our RL agent are the setting of the AS parameter values" => why AS and not Gueant-Lehalle-Fernandez-Tapia approximations which do not include the terminal time T and are considered SOTA and used by the banks?

While this may be regarded as an oversight on our part, the purpose of our experiment was to show that AS model performance improvements can be achieved by using RL to modify its risk aversion parameter and the output spread. We believe the same idea can be applied to more refined AS-inspired models. We intend to explore this in future work.

The conclusions now include the following:

“The original Avellaneda-Stoikov model was chosen as a starting point for our research. Notable refinements of the AS approach have since been proposed, such as Guéant’s (11) closed-form solution to the market maker problem for both single and multiple assets, modelling the mid-price and trades as Brownian movements, and Bergault et al.’s more recent contribution (49), also inspired by the Guéant-Lehalle-Fernandez-Tapia approximations (44). We plan to use such approximations in further tests with our RL approach.”

3) Page 8 => "power to provide optimal quotes in the ideal case" => the AS procedure generates approximations to the optimal quotes and not the optimal quotes. Again, the statement is too strong and should be qualified.

Again, we agree and have modified the text accordingly, to reflect more precisely the underlying goal of the Avellaneda-Stoikov approach, as stated in their original paper:

“leveraging its ability to provide quotes that maximize profit in the ideal case”

4) Page 10 => "The agent is going to repeat the chosen action at every orderbook tick that occurs throughout the time step." Please explain this more thoroughly. Does this mean that, if the action as chosen at the first tick and the order gets executed instantly (and hence the inventory changes), the same action is repeated regardless of the fact that the inventory has changed? If so, why is this not problematic?

The actions (which are set every 5 seconds and repeated at every tick in this period) consist in choosing a value for the risk aversion parameter and one for the skew to be applied to the spread. The AS equations, with these parameters, are used at every tick to obtain new bid and ask quotes, as normal. The only things that stay constant for 5 seconds are the values of the aforementioned parameters, ¡not the quotes! We have added the following clarification to the paper:

“As we shall see shortly, the actions specify two things: the risk aversion parameter in the AS formulas and a skew applied to the prices returned by the formulas. Repeating the action simply means setting these (and only these) two parameters, risk aversion and skew, to the same values for the duration of the 5-second time window. With these parameters thus updated every 5 seconds, fresh bid and ask prices are obtained at every tick, with the latest market information, through the application of the AS formulas.”

5) Why do you opt for discretized large action space instead of simply using a continuous action space and an appropriate RL algorithm, especially given there is a great selection of RL algorithms capable of tackling continuous action spaces? Please elaborate a bit.

We thank the reviewer for this useful suggestion. We have added to the text the following explanation for our design choice for the agents’ action space:

“We chose a discrete action space for our experiment to apply RL to manipulate AS-related parameters, aiming keep the algorithm as simple and quickly trainable as possible. A continuous action space might perform better, but the algorithm would be more complex and the training time greater.”

And in the conclusions we mention a continuous action space as possible future work:

“In the present study we have simply chosen the finite value sets for these two parameters that we deem reasonable for modelling trading strategies of differing levels of risk. This helps to keep the models simple and shorten the training time of the neural network in order to test the idea of combining the Avellaneda-Stoikov procedure with reinforcement learning. The results obtained in this fashion encourage us to explore refinements such as models with continuous action spaces.”

6) Page 10 => "Greater importance was assigned to features that had a larger effect on the reward". How do you precisely determine the effect on the reward? What do you use as labels?

The following explanation has been added to the text:

“The target for the random forest classifier is simply the sign of the difference in mid-prices at the start and the end of each 5-second timestep. That is, classification is based on whether the mid-price went up or down in each timestep. The labels are the state features themselves.”

7) Page 10 => "The agent describes itself by the amount of inventory it holds and the reward it receives after performing actions" => please elaborate on the importance of using reward as part of the state space as it seems unorthodox

The reward driving the agent’s learning, based on which it chooses its actions, is not the quantity reflected in its state. We have added the following, to clarify:

“the reward function is the Asymmetric dampened P&L obtained in the current 5-second time step. In contrast, the total P&L accrued so far in the day is what has been added to the agent’s state space, since it is reasonable for this value to affect the agent’s assessment of risk, and hence also how it manipulates its risk aversion as part of its ongoing actions.”

8) Page 13 => "set {0.01, 0.1, 0.2, 0.9}". How do you obtain the values, are they chosen arbitrarily?

These are simply values chosen to reflect high and low risk-aversion, as noted in the text. This may indeed be regarded as a weakness in our methodology. Perhaps in future work we could obtain a set (or range) of values more systematically. In the meantime, hopefully the experimental results obtained with our current models are deemed of interest.

9) Page 16 => "There are two basic parameters to be determined in our direct Avellaneda-Stoikov model" => why only two, what about the parameter A?

We have followed a more practical approach (more data-grounded), following Aldridge. Our models do not require further parameter specification. The following explanation has been added:

“Note that this approach, following Aldridge’s (7), allows us to estimate the $k$ parameters simply by counting the order arrivals in each time interval, $\pi_{n}$. No further parameter is needed to characterise the asset’s liquidity (such as $A$, if we were to model order arrival rates by the exponential law $\lambda\left( \delta\right)=Ae^{-k\delta}$. as in (2) and (44)).”

Additional comments:

1) Are you acquainted with this GitHub repo? (https://github.com/im1235/ISAC) The authors also seem to be directly controling the risk aversion parameter gamma, i.e., using it as the action.

This is an interesting resource. We did not know it. Thank you. Our programme is written in Java and is probably faster and better suited for use in real environments. We have backtested it with real data. The suggested GitHub repository seems to have simulated data.

**Comments from REVIEWER #3**

Generally, the paper is very hard to read. Some of it is due to the many formatting mistakes, but the organization is also flawed and makes it very hard for the reader to understand the proposed methods and results.

We have striven to make improvements in organisation, formatting, style, and indeed in the content of the paper. It would be very useful to receive more specific comments on the weaknesses that may remain, to help us improve legibility further.

- The double DQN approach is relatively poorly explained, especially when taking into account the fact that this is one of the central parts of the proposed methodology.

We have reworked the overview of DQNs and Double DQNs.

- When considering data and features, the truncation of the ranges to the interval [-1,1] is left somewhat vague - is this a regular machine learning normalization procedure (done only on training data) or something else?

As indicated in the paper, the truncation of the interval of possible state feature values is done simply to limit the state space. The goal, as we mention in the paper, is:

“To derive a manageable number of states from the combinations of all possible feature values.”

It has nothing to do with the normalisation procedures that take place in any neural network (including, of course, those in our models).

- The choices of N=5 ticks for private indicators and N=3 for market indicators seem very arbitrary.

The choice is indeed arbitrary, as is typically the case with parameter tuning in machine learning! We chose the values that “worked for us”. We have added the following to the text:

“In each case, a value of *N* was chosen large enough to provide the agent with a sufficiently rich state space from which to learn, while also small enough that training demands a manageable amount of time and resources.”

- When using the random forest (RF) model for variable selection, it is unclear what the RF classifiers are actually classifying (which output variable and labels were used)?

The following explanation has been added to the text:

“The target for the random forest classifier is simply the sign of the difference in mid-prices at the start and the end of each 5-second timestep. That is, classification is based on whether the mid-price went up or down in each timestep. The labels are the state features themselves.”

- The main benchmark is a static selection of parameters for the AS procedure (the Gen-AS method) - however, the RL based methods are re-trained every 4 hours. A much more fair comparison would be not to re-train the RL methods, or to re-train the Gen-AS method, since in this case it is not only the RL approach that may provide improvement, but also the dynamic choice of AS parameters through time.

This is a good suggestion. In fact, at one point we did experiment with changing the parameters of Gen-AS periodically (obtaining similar results, by the way). However, consider this reasoning, which we have added to the paper:

“They are not recalibrated periodically for the Gen-AS so that their values do not differ from those used throughout the experiment in the Alpha-AS models. If $w$ and $k$ were different for Gen-AS and Alpha-AS, it would be hard to discern whether observed differences in the performance of the models are due to the action modifications learnt by the RL algorithm or simply the result of differing parameter optimisation values. Alternatively, $w$ and $k$ could be recalibrated periodically for the Gen-AS model and the new values introduced into the Alpha-AS models as well. However, this would require discarding the prior training of the latter every time $w$ and $k$ are updated, forcing the Alpha-AS models to restart their learning process every time.”

- A significant issue are the reported statistical results - the Kruskal-Wallis and Mann-Whitney tests are not paired tests (to the reviewer's best knowledge, they assume independent samples). However, the data points themselves (the performance metrics over the test period days) are paired - i.e., the results for the same day are not independent, since they use the same trading data. If paired versions of these tests are used, the results may not hold.

This is an interesting point. We appreciate that the reviewer has brought it up.

We do not quite agree that the samples are related. Indeed, the test data is the same for all models, but the models themselves are very different. Tests for related samples are typically (though not exclusively, of course) used for longitudinal studies. This is not the case here. We feel that to consider that the samples are related would be akin to regarding samples of different people to be related because they are all human beings and have a similar chemical composition, when studying how their bodies react to different drugs (like our models), for instance.

Nonetheless, we have performed tests for related samples on the data (Friedman’s Anova and Wilcoxon Matched Pairs tests) and obtained similar results. We will be happy to report them if the reviewer can further ground his belief that the samples are related.

Other minor issues include:

- the open P&L for the 5-second action time step is defined as Psi(t_i), but the Psi variable is not used in eq. 14 - are these the same quantities?

Yes, they are the same. The formulas have been edited to reflect this.

- Table 8 is somewhat unclear (specifically, the sign comparisons)

A more detailed explanation has been added:

“The “Sign comparison of value differences” side of the table (right) highlights in green (Alpha-AS “better”) the test days for which the respective Alpha-AS models performed worse on Max DD but better on P&L-to-MAP relative to the Gen-AS model, the latter being the more desirable indicator in which to perform well (since maximizing the P&L profile is the central point of the AS method). Conversely, test days for which the Alpha-ASs did worse than Gen-AS on P&L-to-MAP in spite of performing better on Max DD are highlighted in red (Alpha-AS “worse”).”

- reference formatting is not consistent, and some referenced papers appear listed twice in bibliography

Fixed. Thank you.

- additional formatting error such as referencing Section 0, "equations X and X", and several "Error! Reference source not found", capitalization of "python" etc.

Fixed. Thank you.
