# Supplementary material for: A reinforcement learning approach to improve the performance of the Avellaneda-Stoikov market-making algorithm
Source: PLoS One. 2022 Dec 20;17(12):e0277042. doi: 10.1371/journal.pone.0277042 (PMC9767337; doi:10.1371/journal.pone.0277042)
Supplement: Attachment — Submitted filename: ResponseReviewers.docx [file pone.0277042.s003.docx]

Javier Falces Marín

Universidad Politécnica de Madrid

Avenida Complutense, 30 – Dcho C-304.1

28040 Madrid

Spain

J. E. Trinidad Segovia

Section Editor

PLOS ONE

18^th^ September 2022

PLOS ONE *paper Ref.:* **PONE-D-22-10287**

*Title:*

“A reinforcement learning approach to improve the performance of the Avellaneda-Stoikov market-making algorithm”

*Dear Ms. Trinidad Segovia and reviewers,*

*Many thanks, again, for your helpful suggestions to improve this paper. We present below our responses to the second review round, with references to the resulting changes in the manuscript.*

*Kind regards,*

*Javier Falces Marín*

**Responses to the reviewers’ comments**

Comments from the reviewers are on grey background.

Our responses are on white background.

**Comments from REVIEWER #3**

The revised version of the paper addressed the reviewer comments. The author responses to some methodological questions are noted and these points are mostly accounted for by the authors.

We are glad the reviewer deems the changes we have made to the manuscript have answered most of the concerns identified.

The question of the truncation of the interval of possible state feature values remains open, or there seems to be some misunderstanding between the authors and the reviewer. For instance, how are market prices (or actually differences to the mid-price) truncated to the interval [-1,1]? Are they scaled by some scaling parameter beforehand - and what data is this parameter estimated from (in ML literature this is usually done using only the training data)? If not, how much data is lost by only using the price differences with absolute values smaller than 1? Moreover, how are quantities truncated? Also, if the market candle features are "divided by the open mid-price for the candle", does this mean that all of those higher than the mid-price would be would be truncated to 1? The methodology might be more sound than this, but the text simply does not offer answers to these questions.

The reviewer has identified an important point that indeed needed clarification in the paper. We have added a hopefully clearer and more detailed description of the pre-treatment of input data to the model, with brief notes on the rationale supporting it. Particularly, we draw attention to the following paragraph (page 9):

“Together, a) and b) result in a set of $2\times{10}^{d}$ contiguous buckets of width ${10}^{-d}$, ranging from $-1$ to $1$, for each of the features defined in relative terms. Approximately 80 % of their values lie in the interval $[-0.1, 0.1]$, while roughly 10 % lie outside the $\left[ -1, 1 \right]$ interval. Values that are very large can have a disproportionately strong influence on the statistical normalisation of all values prior to being inputted to the neural networks. By trimming the values to the $\left[ -1, 1 \right]$ interval we limit the influence of this minority of values. The price to pay is a diminished nuance in the learning from very large values, while retaining a higher sensitivity for the majority, which are much smaller. By truncating we also limit potentially spurious effects of noise in the data, which can be particularly acute with cryptocurrency data.”

In general, the legibility of the paper is hardly improved, and the revisions in this regards were mostly superficial. The reviewer can point in the directions and give some examples but it is simply impossible to list all of the specific details, and it should be on the authors to check the manuscript in detail.

We are sorry that the reviewer still finds fault with the legibility of the paper. We have made many changes to it, both thanks to the reviewers’ comments and of our own initiative. We believe the paper is much improved, as a result. Nevertheless, we also understand that this reviewer may consider the structural changes to the paper as being lighter than desired. We have strived to improve it as much as possible in this regard, and would wish to do so further, but it is very challenging. The paper touches several fields (market-making and the A-S procedure, reinforcement learning, double DQNs, genetic algorithms), which we feel important to introduce and give some background for, for the benefit of a wider readership. We have made an effort to introduce them all maintaining a logical overall structure throughout the paper, but we recognise that it still also requires an effort on the part of the reader to bring it all together when reading the experimental sections.

To the difficulty we find in further improving the exposition, there is the added risk of undoing what seems to have already pleased the other two reviewers. This discourages us from reorganising the manuscript drastically.

For instance, even after comments about reference formatting, some references have missing publications, years, issues, or even author names (there are many examples, and the authors should take a detailed look into all references and citations). Also, there seems to be a large number of arxiv or SSRN preprints listed for references which are actually published, either as working papers by some institutions or even in peer reviewed journals (and thus the arxiv or SSRN version is not a proper citation).

We have revised the paper and cleaned up these issues.

- there is no need to capitalize terms such as reinforcement learning, profit and loss, Q-learning, Q-value, etc.

This is a matter of stylistic preference. In some works these terms appear capitalised. We have now “decapitalised” them throughout the manuscript to suit the reviewer.

- what does X in the subscript of the features denote, if t is used as the time instance above? Generally, using X as a time index is unusual and might lead to confusion.

X does not denote time directly. It denotes the number of ticks that have passed since the indicator it suffixes took the value we are calling the X^th^ toward the past. We hope our revised text is clearer:

“For each indicator considered, we define N features holding the bucket identifiers corresponding to the current value of the feature (denoted with the suffix X = 0) and its values for the previous N-1 ticks or candles (denoted with the suffixes X = 1, 2, … N-1, respectively).”
